# Tumor-infiltrating B cells as a favorable prognostic biomarker in breast cancer: a systematic review and meta-analysis

**DOI:** 10.1186/s12935-021-02004-9

**Published:** 2021-06-12

**Authors:** You Qin, Fei Peng, Lisha Ai, Shidai Mu, Yuting Li, Chensu Yang, Yu Hu

**Affiliations:** 1 Cancer Center, Union Hospital, Tongji Medical College, Huazhong University of Science and Technology, Wuhan, 430022 China; 2 Institute of Hematology, Union Hospital, Tongji Medical College, Huazhong University of Science and Technology, No. 1277 Jiefang Avenue, Hubei 430022 Wuhan, China

**Keywords:** Tumor-infiltrating B lymphocytes, Breast cancer, Meta-analysis, Prognosis

## Abstract

**Background:**

Tumor-infiltrating B lymphocytes (TIL-Bs) is a heterogeneous population of lymphocytes. The prognostic value of TIL-Bs in patients with breast cancer remains controversial. Here we conducted this meta-analysis to clarify the association of TIL-Bs with outcomes of patients with breast cancer.

**Methods:**

We searched PubMed, Embase, and Web of Science to identify relevant studies assessing the prognostic significance of TIL-Bs in patients with breast cancer. Fixed- or random-effects models were used to evaluate the pooled hazard ratios (HRs) for overall survival (OS), breast cancer-specific survival (BCSS), disease-free survival (DFS), and relapse-free survival (RFS) in breast cancer.

**Results:**

A total of 8 studies including 2628 patients were included in our study. Pooled analyses revealed that high level of TIL-Bs was associated with longer OS (pooled HR = 0.42, 95% CI 0.24–0.60), BCSS (pooled HR = 0.66, 95% CI 0.47–0.85), and DFS/RFS (pooled HR = 0.41, 95% CI 0.27–0.55).

**Conclusions:**

This meta-analysis suggests that TIL-Bs could be a promising prognostic marker for breast cancer. Novel therapeutic strategies for breast cancer treatment could be developed by enhancement of B cell-mediated antitumor immunity.

**Supplementary Information:**

The online version contains supplementary material available at 10.1186/s12935-021-02004-9.

## Introduction

Breast cancer (BC) is a group of malignant diseases arising from the mammary gland [1]. With over 200, 000 new cases each year across the world, it is the most frequent malignancies among women, accounting for 22% of all female cancer types [2]. Despite the recent striking improvements in therapeutic modalities, BC is still the leading cause of cancer-related death worldwide among women [2, 3].

The comprehensive understanding of the immune responses in cancer initiation and progression has only advanced during recent years [4], even though tumor-infiltrating immune cells were reported by Rudolf Virchow over 100 years ago in 1863 [5, 6]. The presence of tumor-infiltrating lymphocytes (TILs) at diagnosis is correlated with improved survival after adjuvant systemic therapy and pathological complete responses to neoadjuvant chemotherapy in BC [7–10]. These findings have provided substantial evidence of the protective potential of lymphocytes within tumors.

B cell is one of the main immune components, occupying a central position in forming the tumor immune microenvironment [11, 12]. Both antitumor and tumor-promoting functions of B cells have been reported in tumor immunity and immunotherapy [11]. Evidence accumulating in the late 1990 s facilitated a widespread acceptance of B cell-mediated protumor functions [13]. However, more recent publications have shown that B cells employ a protective rather than a detrimental property in human solid tumors [14–18]. Intriguingly, there exists considerable controversy over the prognostic impacts of TIL-Bs in different cancer types or subtypes including BC [19–21].

Growing evidence has revealed that TIL-B is a positive prognostic indicator in human BC. For example, in inflammatory breast cancer (IBC), CD20 + TIL represents a prognostic factor of better outcomes [22]. Similarly, a separate study shows that high levels of TIL-Bs are associated with improved prognosis of BC patients [23]. A B-cell metagene has been shown prognostic value for node-negative BC with high proliferative activity [24]. This was followed by an immunochemistry (IHC) study associating an increased total number of TIL-Bs with significantly better breast cancer-specific survival (BCSS) in basal-like, estrogen receptors negative (ER-), and HER2 + cancer patients [25]. In contrast to these studies, Miligy et al. have shown that preinvasive ductal carcinoma in situ associated with increased TIL-Bs had poorer recurrence-free survival [26].

Clarifying the prognostic role of TIL-Bs could improve the understanding of B cells in antitumor responses, with help of developing more effective immunotherapies for BC. To this end, we conducted the present meta-analysis, showing that higher densities of TIL-Bs were significantly associated with prolonged overall survival (OS), BCSS, disease-free survival (DFS), and relapse-free survival (RFS) in BC patients. This finding suggests that B cells function in antitumor response and might be a promising source for novel therapeutic strategies for BC patients.

## Methods

### Search strategy

We conducted this meta-analysis following the search strategies [27] and the Preferred Reporting Items for Systematic Reviews and Meta-Analyses (PRISMA) guidelines [28]. PubMed, Embase, and Web of Science were searched without limits to identify all relevant studies until October 2020. Detailed search strategy was shown in Additional file 1. Keywords used in the search process are as follow: “B cells” (e.g., “tumor-infiltrating B cells” “TIL-Bs” “B lymphocytes” ”intratumoral B lymphocytes”), “prognosis” (e.g., “survival” “mortality” “outcome” “progression” “recurrence” “metastasis”) and “breast cancer” (e.g., “breast carcinoma” “carcinoma of the breast” “breast ductal carcinoma”). We also explored references from previously published meta-analyses and reviews to find more potential studies.

### Selection and exclusion criteria

Studies meeting the following criteria were included: (i) studies reported the association between TIL-Bs and prognostic parameters in breast cancer; (ii) HR and 95% confidence intervals (CI) were reported or could be reconstructed by p values and other data reported; (iii)studies applied IHC as a detection method and used CD20 as a molecular marker of B cells; (iv) the sample size of studies was greater than 50. Exclusion criteria were: (i) Conference abstracts, reviews, case reports, letters, animal trials, etc.; (ii) studies without specific or sufficient data concerning breast cancer or TIL-Bs; (iii) studies were excluded if the sample size < 50, to reduce publication bias caused by small sample size; (iv) Only the most recent publication was enrolled in this meta-analysis if one patient cohort were investigated by several studies.

### Data extraction

Data extraction was performed by two independent reviewers (You Qin and Lisha Ai). The disagreement was resolved by discussion with the other investigator (Fei Peng) until the two reviewers reached a consensus or by consulting experts if necessary. A data abstraction form was predefined with key elements such as the first author’s name, year of publication, country of publication, types of breast cancer, sample size, patient age, follow-up duration, cut-off value, and survival data including OS, BCSS, DFS, and RFS. BCSS was defined as the time between breast cancer diagnosis and death due to breast cancer, while OS was the period between diagnosis and death due to all causes (including breast cancer). DFS was the period after curative treatment when no disease can be detected. RFS was defined as the interval between the date of initial treatment and the data of recurrence or death from any cause; We used HR and 95% CI directly If they were reported in the study; Otherwise, we reconstructed the data from Kaplan–Meier curves and p values using the software Engauge Digitizer version 4.1 [29].

Quality of evidence was assessed by the Newcastle-Ottawa Scale (NOS) [30]. Based on this scale, each study is evaluated on several domains, including the comparability of groups, the selection of participants, the ascertainment of outcomes of interest, et al. Studies can be scored for each domain. A study is considered high-quality if there is a score of 6 or more (the maximum score is 9).

### Statistical analysis

This meta-analysis was conducted using STATA statistical software (version12.0; Stata Corporation, College Station, TX). The prognostic effects of TIL-Bs in breast cancer were evaluated by HRs and 95% CIs. HR was considered as the risk ratio between patients with rich TIL-Bs versus those with low TIL-Bs. Thus, an HR < 1 implied TIL-Bs as a good prognosis, whereas an HR > 1implied TIL-Bs as a poor prognosis. We assessed the heterogeneity by using the *χ*^2^-based Q test and the *I*^2^ test. Statistically significant heterogeneity was defined as *I*^2^ was greater than 50 % and/or *P* value was less than 0.05. Then sensitivity analysis and subgroup analysis were performed to assess the source of heterogeneity. Publication bias was also quantified by Begg’s tests and Funnel plot.

## Results

### Search results

**Fig. 1:**
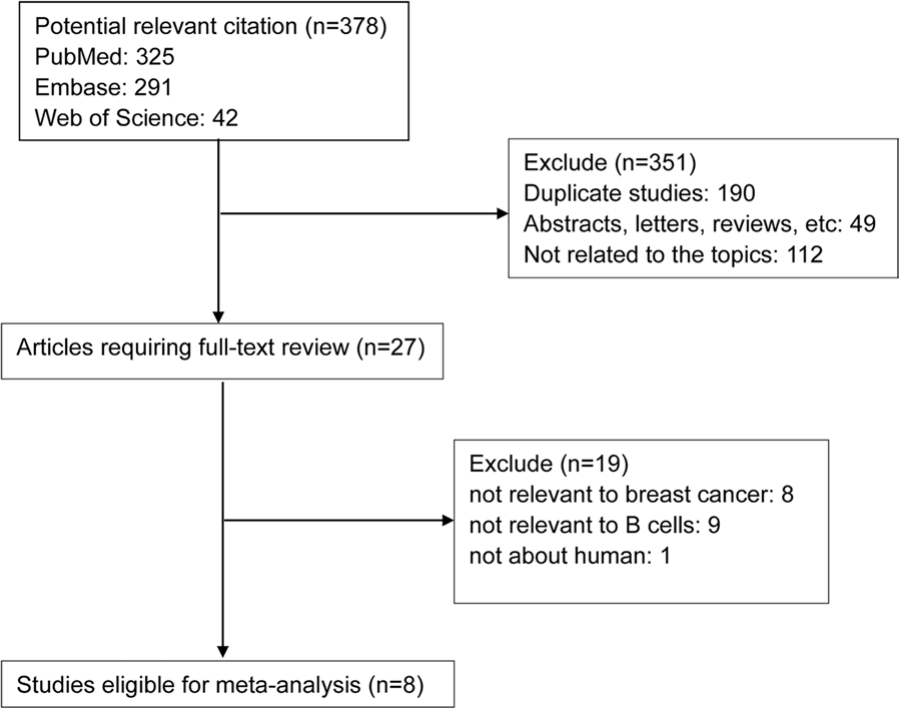
Flow chart of study selection

A total of 378 studies were identified after the initial search. After exclusion of the duplicate records (n = 190); letters, conference abstracts, reviews, etc. (n = 49); and the studies not relevant to topics (n = 112), the remaining studies (n = 27) were left for full-text evaluation. Then 19 full-text articles were removed for reasons: no available data, no specific data concerning breast cancer or B cells, and overlapped patients. At last, 8 studies between 2012 and 2020 were enrolled in the current meta-analysis [22, 23, 25, 31–35]. The process of the literature search was shown in Fig. 1. One research reported two cohorts of breast cancer patients (TNBC cohort and HER2 + cohort), thus we treated it as two studies [31].

### Study characteristics

The total sample size was 2,628, ranging from 102 to 1214. All these studies were retrospective analysis of breast cancer. Nine studies enrolled > 100 patients and one study even enrolled > 1000 patients [25]. All these studies were retrospective analysis of breast cancer. These studies were single-center studies, and conducted in the UK [25, 32], Belgium [31], France [23], Singapore [35], Algeria [22], and China [33, 34], which evaluated several types of breast cancer, including TNBC, HER2 + breast cancer, invasive ductal carcinoma of the breast, and breast ductal carcinoma in situ. The cutoff value of TIL-Bs ranged from 1 to 5.5% or 1 cell to 115 cells. Six studies reported the prognostic value of TIL-Bs on OS [22, 31, 33–35], two studies showed the association between TIL-Bs and BCSS [25, 32], six studies evaluate the prognostic effects of TIL-Bs on DFS [22, 31, 33–35], and one study reported RFS [23]. Seven studies reported HR and 95% CI directly in the original paper. Eight studies had a NOS score ≥ 6. The characteristics of these studies were shown in Table 1.

**Table 1:** Characteristics of each study

First author	Year	Country	Cancer types	Sample size	Cut off	Median age	Median follow-up (month)	Research type	Survival analysis	HR type	NOS
Mahmoud	2012	UK	Primary invasive BC	1214	1 cell	55	250	Retrospective analysis	BCSS	Reported	6
Garaud 1	2019	Belgium	HER2 + BC	136	0.055	NR	120	Retrospective analysis	OS, DFS	Reported	8
Garaud 2	2019	Belgium	TNBC	113	0.0275	NR	120	Retrospective analysis	OS, DFS	Reported	8
Arias-Pulido	2018	Algeria	IBC	221	0.01	NR	96	Retrospective analysis	OS, DFS	Estimated	5
Yeong	2018	Singapore	TNBC	269	0.05	55	97	Retrospective analysis	OS, DFS	Reported	7
Xu	2018	China	Invasive ductal BC	102	115 cells	48	60	Retrospective analysis	OS, DFS	Reported	6
Mohammed	2013	UK	Primary operable ductal invasive BC	338	5 cells	NR	164	Retrospective analysis	BCSS	Reported	8
Boissière-Michot	2020	France	TNBC	105	NR	NR	120	Retrospective analysis	RFS	Reported	7
Yu	2013	China	BC	130	NR	NR	NR	Retrospective analysis	OS, DFS	Estimated	6

*BC* breast cancer; *TNBC* triple-negative breast cancer; *IBC* inflammatory breast cancer; *HER2* human epidermal growth factor receptor-2; *NR* not reported; *BCSS* breast cancer-specific survival; *OS* overall survival; *DFS* disease-free survival; *RFS* relapse-free survival; *HR* hazard ratio; *NOS* Newcastle-Ottawa Quality Assessment Scale

### TIL-Bs and survival of BC patients

**Fig. 2:**
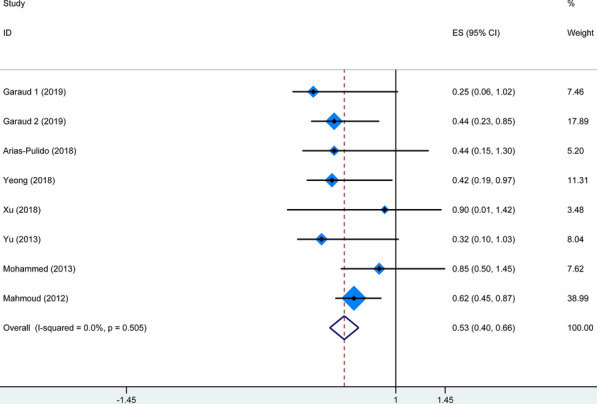
Forest plot for the association of TIL-Bs with OS and BCSS in BC patients

**Fig. 3:**
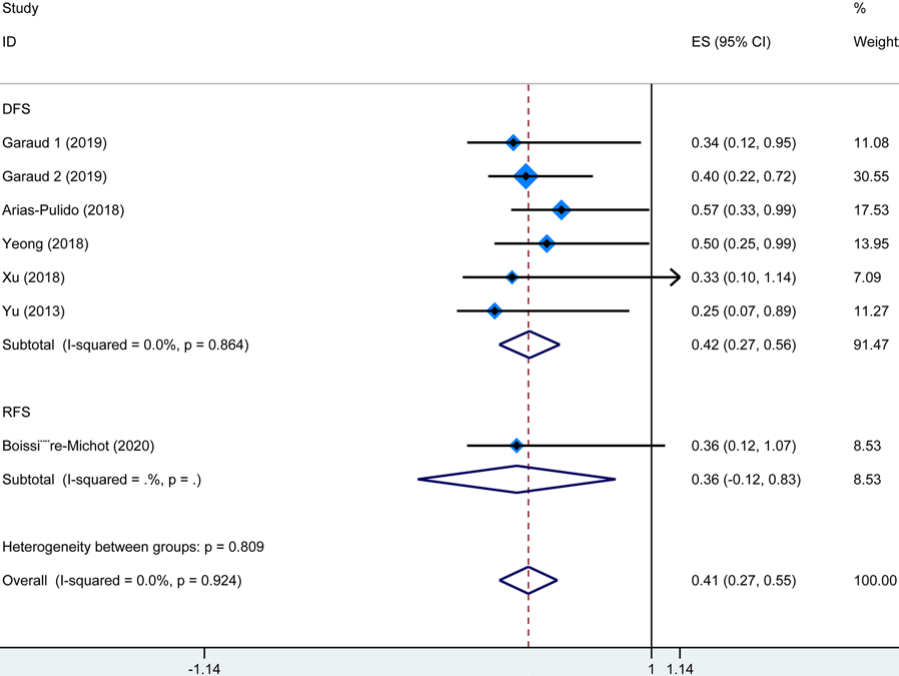
Forest plot for the association of TIL-Bs with DFS/RFS in BC patients

Nine studies explored the association between TIL-Bs and survival of patients with BC. Figure 2 summarized HR for OS in 6 studies (HR = 0.42, 95% CI 0.24–0.60) and BCSS in two studies (HR = 0.66, 95% CI 0.47–0.85), and there was no heterogeneity among the studies (*I*^2^ = 0%, P_heterogeneity_ = 0.783; and *I*^2^ = 0%, P_heterogeneity_ = 0.385, respectively). In addition, as shown in Fig. 3, the combined results of 7 studies showed higher levels of TIL-Bs were associated with longer DFS/RFS (HR = 0.41, 95% CI 0.27–0.55) without significant heterogeneity (*I*^2^ = 0%, P_heterogeneity_ = 0.924). These results suggested that a higher TIL-Bs level was significantly correlated with better survival in patients with BC.

### Sensitivity analysis

**Fig. 4:**
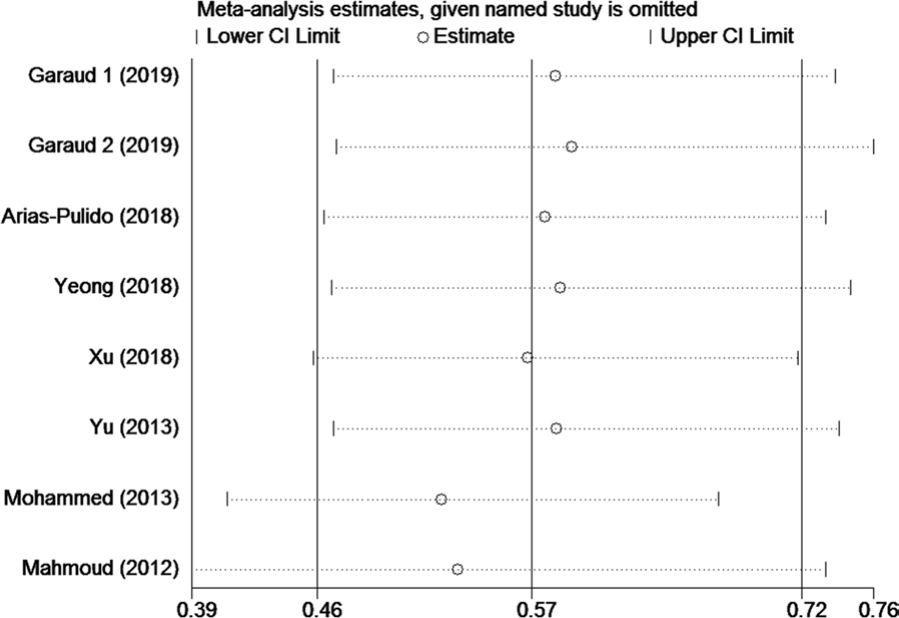
Sensitivity analysis of the enrolled studies

Sensitivity analyses were then carried out to assess the influence of every single study on the pooled HRs. As shown in Figs. 4, the pooled HRs were not significantly altered by any individual study, indicating the stability of our results.

### Publication bias

**Fig. 5:**
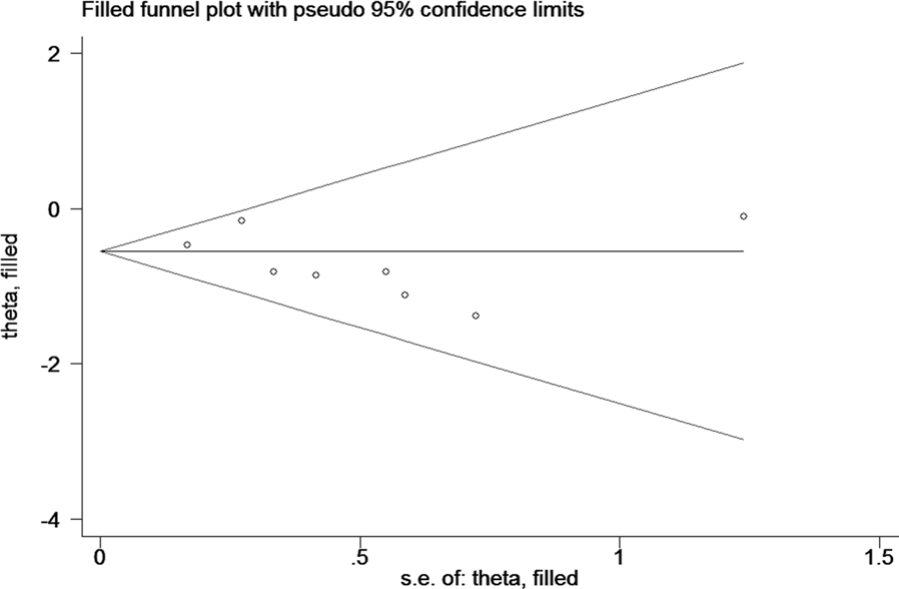
Funnel plot with trim and fill

Begg’s funnel plots were conducted to explore the publication bias of the included studies (Fig. 5). The results show that there was no significant asymmetry, and the rank correlation tests were not significant (*P* = 0.175), indicating no significant evidence of publication bias.

## Discussion

The initiation and development of breast cancer are derived not only by the genetic abnormalities but also by interplays between cancer cells and their surrounding tumor microenvironment (TME) [36], which is comprised of a variety of tissues and cell types, including blood vessels, fibroblasts, extracellular matrix, and immune cells [37]. Accumulating evidence has shown that the infiltrates of lymphocytes, such as T cells, dendritic cells, macrophages, and B cells, occupy a central position in promoting or suppressing tumorigenesis and cancer progression [37]. Thus, targeting and remodeling the tumor immune microenvironment is a promising strategy to develop novel immunotherapies for cancers. It has been well recognized that cytotoxic CD8 + T cells confer antitumor immunity [38]. Although both T cells and B cells take part in tumorigenesis, relatively limited studies have focused on B cell-mediated antitumor or protumor responses [39]. Additionally, both positive and negative prognostic significance of TIL-Bs has been reported in human solid tumors [20, 21, 40], including BC, resulting in differing opinions concerning whether cancer immunotherapies should be designed to promote or suppress B cells [21].

To make sense of the current controversial findings, we conducted the present systematic review and meta-analysis to verify the contribution of intratumor B cells to the prognosis of BC patients. We comprehensively searched the databases (e.g., PubMed, Embase, and Web of Science) and finally included 8 eligible cohort studies addressing the association of TIL-Bs with the survival of BC patients utilizing B cell marker CD20 tested by IHC. The pooled analysis showed that high levels of TIL-Bs were significantly correlated with the favorable OS, BCSS, DFS, and RFS of patients with BC, suggesting that B cells within tumor tissue might exert antitumor immunity.

Although both antitumor and protumor-promoting activities of TIL-Bs have been reported in tumor immunity and immunotherapy [11], more recent findings have demonstrated that B cells employ a protective rather than a detrimental property in human solid tumors [14–18], which can properly explain the positive prognostic value of TIL-Bs in BC shown in our meta-analysis. TIL-Bs exert antitumor functions via direct tumor-killing effects, antigen presentation, antibody production, cytokine secretion, and other activities [41–43]. First, it has been reported that activated B cells have the potential to directly kill tumor cells via antibody-independent mechanisms [44–49], which provides the possibility for adoptive cellular therapy based on B cells. Tao and his colleagues reported that effector B cells directly killed tumor cells via the Fas/FasL and CXCR4/CXCL12 pathways, and the killing activities can be improved by IL-2 and inhibited by IL-10 [44–49]. Second, B cells have been proved to function as antigen-presenting cells (APCs) to engage with T cells, triggering anti-cancer responses. Human B cells can efficiently present peptides to CD4 + T cells after activated by CD40 ligand and pulsed with tumor antigens [50]. Colluru and colleagues successfully applied B cells as a tool to present tumor DNA to CD8 + T cells as a vaccine, which elicited an antitumor effect in vivo [51]. In another report, B cells were reported to infiltrate into the brain tumor site, where they promoted T cell-induced tumor killing as APCs [52]. Third, Multiple lines of evidence have demonstrated that B cells within tumor-related tertiary lymphoid structures can be converted to antibody-producing memory B cells or plasma cells after exposure to tumor-associated antigens [53, 54], thus producing antitumor antibodies within tumor sites and meditating tumor-killing effect [11].

Meanwhile, intratumoral B cells and antibodies may also act as immunosuppressive players [55], thereby driving tumor growth. B cells with protumor properties are mostly described as regulatory B cells (Bregs), which have been identified by cell surface markers and secretion of cytokines, functioning by suppressing immune responses and promoting tumor progression [56–60]. Clinical cohort studies report that Breg enhancement in the TME predicts poorer outcomes in cancer patients [58, 61, 62], suggesting that Bregs might actively participate in tumor immune escape. More specific markers regarding different B cell subsets deserve extensive study to precisely investigate the role of various B cell subpopulations in the prognosis of BC patients.

Some limitations should be noted in this systematic review and meta-analysis, requiring careful interpretations of the results. First, the cut-off value for defining a high level of TIL-Bs varied among the enrolled studies. Second, our results may somewhat overestimate the prognostic significance of TIL-Bs in BC patients, because most of the included studies reported positive results. Third, all the enrolled studies were retrospective, and thus well-designed prospective studies are needed to further investigate the association between TILs-B and survival of breast cancer. Finally, only 4 out of 8 included studies have provided the molecular subtypes of BC, leading to insufficient analysis of the prognostic impact of TIL-Bs in different specific subtypes. Despite the above limitations, the present meta-analysis supports TIL-Bs as a biomarker for predicting survival in BC patients due to the convincible results as well as the easy-going detection method IHC of TIL-Bs.

## Conclusions

To our best knowledge, this is the first meta-analysis to pool analyze the prognostic role of TIL-Bs in BC patients. Here, we comprehensively searched databases for relevant studies, and included8 cohort studies with a total of 2,628 patients for the current meta-analysis, concluding that BC patients with elevated TIL-Bs have prolonged survival, which could help to stratify the patients into different categories and select personalized cancer therapies. More importantly, novel anti-BC therapeutic strategies would be developed by enhancement of B cell-mediated antitumor immunity. More multicenter prospective cohort studies should be conducted to further verify the role of TIL-Bs in different molecular subtypes of BC.

## Supplementary Information

**Additional file 1.** Search strategy

## Data Availability

Please contact the author for data requests.

## Abbreviations

BC: Breast cancer
TILs: Tumor-infiltrating lymphocytes
TIL-Bs: Tumor-infiltrating B lymphocytes
TNBC: Triple-negative breast cancer
IBC: Inflammatory breast cancer
IHC: Immunochemistry
BCSS: Breast cancer-specific survival
ER: Estrogen receptor
PR: Progesterone receptor
HER2: Human epidermal growth factor receptor-2
OS: Overall survival
DFS: Disease-free survival
RFS: Relapse-free survival
HR: Hazard ratio
TME: Tumor microenvironment
APCs: Antigen-presenting cells
Bregs: Regulatory B cells
