# Supplementary material for: Tumor-infiltrating B cells as a favorable prognostic biomarker in breast cancer: a systematic review and meta-analysis
Source: Cancer Cell Int. 2021 Jun 12;21:310. doi: 10.1186/s12935-021-02004-9 (PMC8199375; doi:10.1186/s12935-021-02004-9)
Supplement: Supplementary file 1 — Additional file 1. Search strategy [file 12935_2021_2004_MOESM1_ESM.docx]

Pubmed：

(((((breast cancer) OR (breast carcinoma)) OR (carcinoma of the breast)) OR (breast ductal carcinoma)) AND (((((((prognosis) OR (survival)) OR (mortality)) OR (outcome)) OR (progression)) OR (recurrence)) OR (metastasis))) AND (((((B cells) OR (tumor-infiltrating B cells)) OR (TIL-Bs)) OR (B lymphocytes)) OR (intratumoral B lymphocytes))

Embase：

| (breast cancer/ or intraductal carcinoma/ or carcinoma in situ/ or breast carcinoma/) and (prognosis/ or cancer prognosis/ cause specific survival/ or survival/ or overall survival/ or cancer free survival/ or disease free survival/ or survival analysis/ or progression free survival/ or cancer specific survival/ or event free survival/ or disease specific survival/ or cancer survival/mortality/ or cancer mortality/ or all cause mortality/ clinical outcome/ progression free survival/ recurrent disease/ metastasis/) and (B lymphocyte/) |  |
| --- | --- |

Web of Science:

((breast cancer) OR (breast carcinoma) OR (carcinoma of the breast) OR (breast ductal carcinoma)) AND ((B cells) OR (tumor-infiltrating B cells) OR (TIL-Bs) OR (B lymphocytes) OR (intratumoral B lymphocytes)) AND ((prognosis) OR (survival) OR (mortality) OR (outcome) OR (progression) OR (recurrence) OR (metastasis))
